# GPA: A Microbial Genetic Polymorphisms Assignments Tool in Metagenomic Analysis by Bayesian Estimation

**DOI:** 10.1016/j.gpb.2018.12.005

**Published:** 2019-04-23

**Authors:** Jiarui Li, Pengcheng Du, Adam Yongxin Ye, Yuanyuan Zhang, Chuan Song, Hui Zeng, Chen Chen

**Affiliations:** 1 Beijing Key Laboratory of Emerging Infectious Diseases, Institute of Infectious Diseases, Beijing Ditan Hospital, Capital Medical University, Beijing 100015, China; 2 Center for Bioinformatics, State Key Laboratory of Protein and Plant Gene Research, School of Life Sciences, Peking University, Beijing 100871, China

**Keywords:** Next-generation sequencing, Pool-seq, Bayesian model, Metagenomics, Genetic polymorphisms

## Abstract

Identifying antimicrobial resistant (AMR) bacteria in **metagenomics** samples is essential for public health and food safety. **Next-generation sequencing** (NGS) technology has provided a powerful tool in identifying the genetic variation and constructing the correlations between genotype and phenotype in humans and other species. However, for complex bacterial samples, there lacks a powerful bioinformatic tool to identify **genetic polymorphisms** or copy number variations (CNVs) for given genes. Here we provide a Bayesian framework for genotype estimation for mixtures of multiple bacteria, named as Genetic Polymorphisms Assignments (GPA). Simulation results showed that GPA has reduced the false discovery rate (FDR) and mean absolute error (MAE) in CNV and single nucleotide variant (SNV) identification. This framework was validated by whole-genome sequencing and **Pool-seq** data from *Klebsiella pneumoniae* with multiple bacteria mixture models, and showed the high accuracy in the allele fraction detections of CNVs and SNVs in AMR genes between two populations. The quantitative study on the changes of AMR genes fraction between two samples showed a good consistency with the AMR pattern observed in the individual strains. Also, the framework together with the genome annotation and population comparison tools has been integrated into an application, which could provide a complete solution for AMR gene identification and quantification in unculturable clinical samples. The GPA package is available at https://github.com/IID-DTH/GPA-package.

## Introduction

Bacterial antimicrobial resistance is considered as “one of the biggest threats to global health, food security, and economic development today” by the World Health Organization (WHO). The effective prevention and treatment of infections caused by bacteria require increasingly greater monitoring and prevention activities [1]. Traditional bacterial monitoring methods by the culture technology followed with the classification by genotyping such as multi-locus sequence typing (MLST) [2] and multi-locus VNTR analysis (MLVA) [3], and pulsed-field gel electrophoresis (PFGE) [4], have been used for more than thirty years to determine causative agents in outbreaks and track the epidemiological trends [5]. However, at present, the culture technology and large genomic diversity within the species limited the development of these monitoring methods. For example, only 126 bacteria have available typing schema for MLST test [6], [7], [8]. This limitation resulted in a 20% shortfall of global outbreaks that could not be adequately illustrated [9]. Improving the genotyping method is an urgent need for better monitoring and epidemiological surveillance.

Next-generation sequencing (NGS) technology has provided immense genotyping data for observing rare and low-frequency genetic variations in complex samples for precision medicine [10]. In humans and other mammals, these data are well employed to observe molecular variations such as copy number variation (CNV) and single nucleotide variant (SNV) [11]. A series of bioinformatic tools have been developed, such as BreakSeek [12], STRiP [13], SVM^2^ [14], LUMPY [15], and inGAP-sv [16]. Among them, STRiP is well known for CNV detection [13], while GATK [17] and SAMtools [18] tools are used for SNV identification. However, the model fitting process of these tools was based on a diploid genome, such as human or mouse genome. Unlike mammals, the clinical samples are complex samples with a large amount of bacteria presenting a large genomic diversity, even within the same species [19]. These tools thus showed a large bias in quantifying the proportion of genes from single species. Also, some metagenomics analysis tools have also been developed to investigate the molecular variations in complex samples, to measure bacterial diversity and abundance, or to identify functional changes in microbial communities under a species level [20]. However, given the lack of an efficient algorithmic model to quantify the identified genes in complex samples, we have few bioinformatics tools to continuously observe the dynamic and potential quantitative changes in a given bacterial population.

The Bayesian model is a widely used algorithm to estimate bacterial diversity and abundances in complex NGS data samples for its strong capability to estimate genotypes in spite of sequencing errors [21], [22]. These errors may be generated for several reasons [13]: molecular libraries contain chimeric molecules that may be misidentified as structural variants [23]; read depths vary across the genome in ways that they also vary among different sequencing libraries [24]; and alignment algorithms are misled by the tandem repeats in the genome [25]. GATK, developed by Broad Institute, has been applied to reduce the impact of the errors in human and mouse sequencing data. For complex bacterial samples, this software may be used with a polyploidy algorithm to estimate SNV proportions [17]. However, GATK has rarely been evaluated in these samples. In addition, a Bayesian model pipeline for estimation of CNVs in bacteria is still lacking, with the exception of Breseq that can only be used for one genome at a time [26].

Here, we have developed the Genetic Polymorphisms Assignments (GPA), a Bayesian framework for genotyping multiple bacterial in mixed samples, including a genome annotation pipeline. To evaluate the accuracy of GPA, we compared Pool-seq data and individual genomic data of *Klebsiella pneumonia*, and found that GPA could (i) detect all SNVs and CNVs in Pool-seq data, and (ii) calculate an accurate frequency of each known allele of target genes, which were identified by individual genomic data. We further demonstrated its capabilities through a consecutive analysis on the frequency of the *tolC* gene, as well as *rmpA* and *rmpA2,* which we previously reported in CR-HvKP, showing sequence analysis consistent with phenotypic changes over two years. This software package can also be used to monitor antimicrobial resistant genes in metagenomics data for a specific pathogen.

## Results

### Performance evaluation by simulation studies

#### Overview of the GPA package

**Figure 1:**
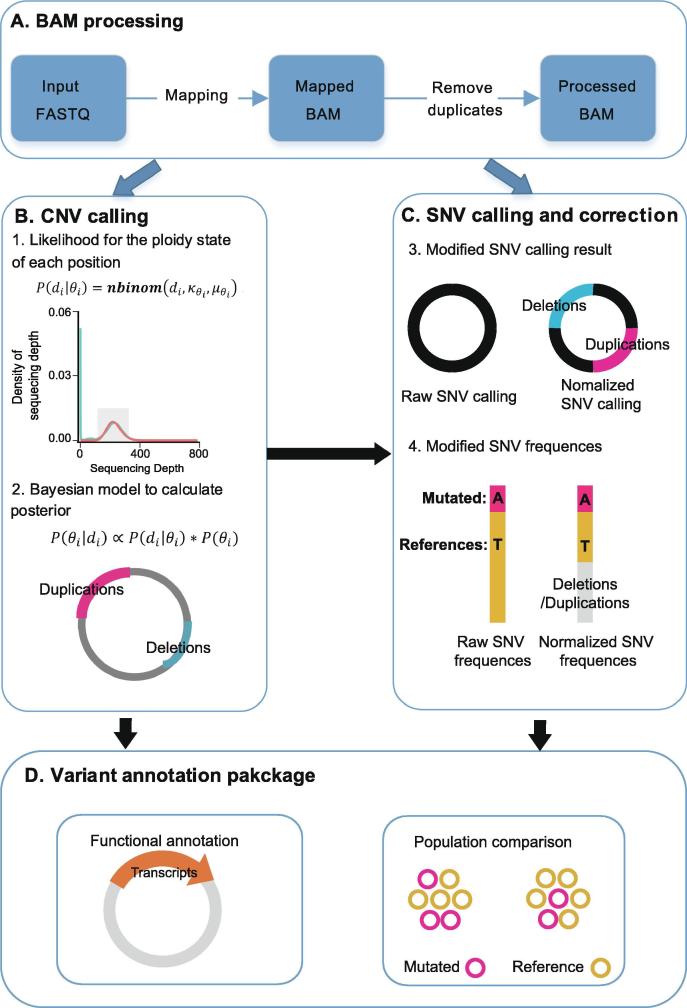
**Schematic overview of the GPA package** **A.** The process of data mapping. **B.** The CNV calling model: the depth in each position is analyzed (upper panel) and predicted for its ploidy number with a Bayesian model (down panel). The pink and blue lines in the circle indicate duplication and deletion regions, respectively. **C.** The SNV calling and correction model: the ploidy analysis result and processed BAM file are used as input for SNV calling and identification with GATK UnifiedGenotyper. The allele fraction is corrected using the CNV identification result. The fractions of the alternate and reference alleles are indicated in red and yellow, respectively. The gray bar indicate deletion regions. **D.** The CNV and SNV calling results are annotated using the reference genes for functional prediction and further population analysis. The orange arrow represents a transcript in the genome. The mutants in the population are drawn with yellow or pink circles. CNV, copy number variation; SNV, single nucleotide variation.

The workflow of the GPA package is shown in Figure 1. First, we processed the BAM file with the standard GATK pipeline: raw reads were mapped with BWA-mem and duplicates were removed with Picard (Figure 1A). Then, the BAM files were processed in the novel CNV calling pipeline model. Using a Bayesian framework, GPA analyzes the coverage depth at each position and predicts its ploidy type (Figure 1B). The results of this ploidy assignment were used as input for the further identification of SNV with GATK UnifiedGenotyper calling (Figure 1C). The total CNV and SNV results were then mapped to reference genes and annotated for functional prediction and population analysis (Figure 1D). This pipeline serves as a complete toolkit for genotype assignments in pooled bacterial sequence data. The GPA package is available at https://github.com/IID-DTH/GPA-package.

#### GPA model applied to a simple CNV example

**Figure 2:**
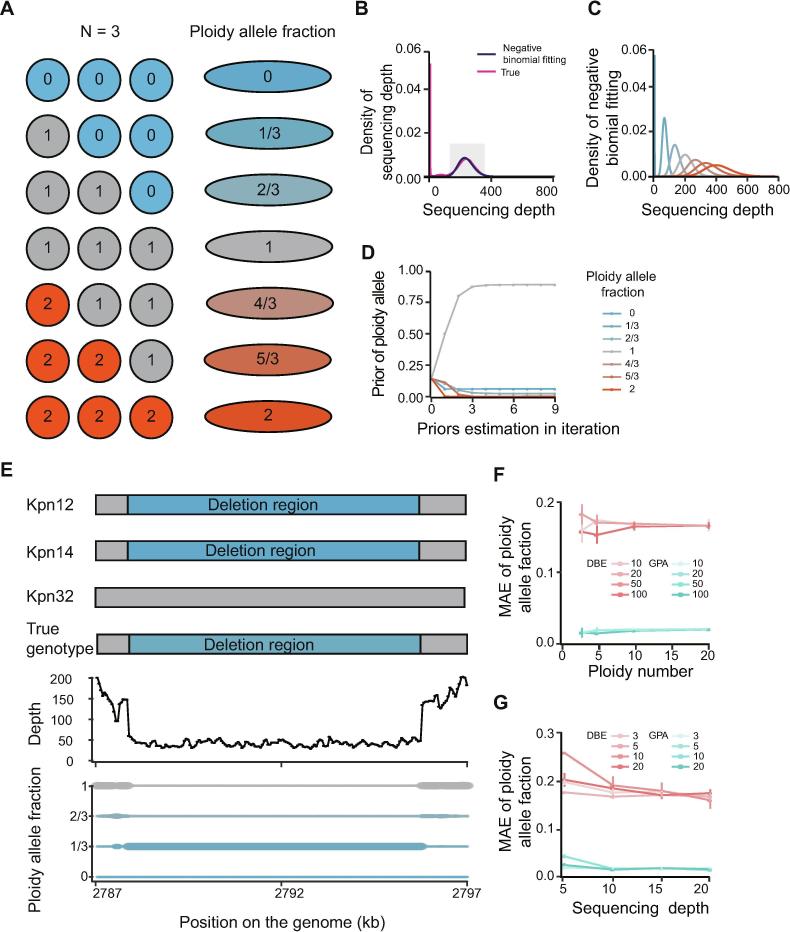
**CNV model in the GPA package** **A.** Diagram of how ploidy allele fraction is calculated using an example ploidy (*n* = 3). Blue indicates deletion, and red indicates duplication. “1″ is a normal stage with a mixture of 3 bacteria. **B.** The negative binomial distribution fitting the main distribution curve of coverage depth for each nucleotide site. The red line represents the real depth distribution in a simulation using the Pool-seq data from 3 genomes. The black line represents the fitted negative binomial distribution, and the shadow region represents the main peak. **C.** Read depth modeled by a negative binomial distribution with different allele fractions. See panel A for color codes. **D.** Prior estimation using iterations. **E.** An example of GPA used to identify Kpn deletion regions in Pool-seq data. The individual genome shotgun sequencing data for Kpn12 and Kpn14 have a deletion in the genome indicated by blue rectangles. The size of this deletion in the bottom panel represents the estimated posterior for different allele types. **F.** MAE calculated with different ploidy numbers using GPA (green) and traditional DBA (red). The color gradient of the lines (from light to dark) represents the coverage depth in the model of 10×, 20×, 50×, and 100×, respectively. **G.** Evaluation of GPA performance in low sequencing depth. MAE was calculated with different coverage depths using GPA (green) and traditional DBA (red). The color gradient of the lines (from light to dark) represents the number of ploidies in the model, which are 3, 5, 10, and 20, respectively. MAE, mean absolute error.

To better illustrate the GPA model when used in a CNV study, we used a mixed dataset consisting of three randomly selected *Klebsiella pneumoniae* genomes (Kpn12, Kpn14, and Kpn32) as Pool-seq data to evaluate the model, posterior of different genotypes, and major allele assignment (Figure 2A–E). Considering the previous bioinformatics tools preferred to applying in mammals with a diploid sample, we used a mixture with three samples as a simple model, which also tends to be a common occurrence in metagenomes. We used this model to show deletion, normal, or insertion states with a 0, 1, or 2 in each reference genome position (Figure 2A). Seven allele types were thus presented for each position in Pool-seq data, including 0, 1/3, 2/3, 1, 4/3, 5/3, and 2. (Figure 2A).

The Pool-seq data were aligned to the reference genome, and fitted the reads depth with a negative binomial distribution curve and estimated its parameter κ (Figure 2B). Using this parameter, we calculated the likelihood value of each modeled negative binomial distribution in each possible allele type (Figure 2C). Next, the posterior were estimated by multiple iterations, and we observed that the priors converged quickly in 3 iterations (Figure 2D). The major allele assignment was displayed with 3 individual genome structures and compared with mixed data. We found that both Kpn12 and Kpn14 genomes had a ∼170-bp deletion, while Kpn32 was in normal stage. This result was consistent with the observation in mixed data, which was assigned with the peak value of allele type according to the posterior (Figure 2E).

#### Performance evaluation of CNV analysis by simulation studies

We expanded the mixture analysis from 3 genomes to multiple genomes (>3). We used genomic data from 2009 and 2013 from 44 *K. pneumoniae* isolates as a control, with shotgun reads having an average depth of 150× as a simulated data pool. 24 sample data were generated by constant coverage (5, 10, 15, 20, 50, and 100×, respectively) from 3, 5, 10, and 20 randomly selected samples. To improve robustness, we repeated to generate three replications for each sample data. The mean absolute error (MAE) of the control was used to measure the performance of different estimation methods.

We compared the performance of GPA with the previous depth-based estimation (DBE) approach. Both approaches readily identified deletions and duplications that occurred in most strains, however, more errors were introduced when deletions or duplications occurred in only a few strains (*i.e.*, the allele frequency was near to the peak) (Figure S1). In this condition, the GPA method had fewer false positive results in the detection of deletions or duplications. The average MAE was 0.02 for GPA and 0.2 for DBE. To evaluate the effect of depth, we simulated an average depth of 10, 20, 50, and 100×. The results showed that the GPA method was more tolerant of low depth datasets (Figure 2F). For depths of more than 20×, the detection was relatively stable. To evaluate the effect of ploidy number in low depth datasets, we simulated ploidies of 3, 5, 10, and 20 under the depth of 20×. The MAE was relatively dispersed when the ploidy number was small (Figure 2G). The GPA method was more robust compared to the DBE method in both low depth and small ploidy number. In total, the GPA method detected polymorphisms more accurately.

#### GPA model with a simple SNV example

**Figure 3:**
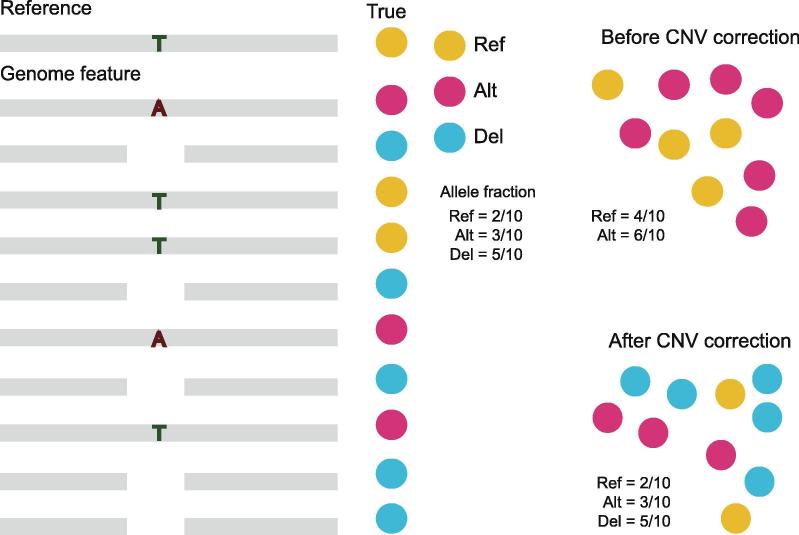
**SNV correction model in the GPA package** Genomic feature of 10 sequencing reads in a given genomic site. The yellow circles represent sites on this genome that have the same allele as the reference genome “T”, the red circles represent alternative alleles, for example “A”, and the blue circles represent deletion sites. Ref, reference allele; Alt, alternative allele; Del, deletion allele.

To better illustrate the GPA model when used in an SNV study, we presented an example of SNV with 10 ploidies (Figure 3). Notably, in this model, five genomes had a deletion in the middle of the sequence. Traditional SNV calling tools always ignore to correct the mutation frequencies, when the mixed samples have deletions or duplications in the given genome site. In this example, there were five deletion alleles in a particular position, two reference alleles and three alternative alleles; the traditional SNV caller reported four reference alleles and six alternative alleles, determining a ploidy level of 10 in this position. The detected alternative allele fraction (6/10) would be higher than the true state (3/10), leading to an incorrect conclusion. In contrast, the duplication would lead to a relatively lower estimate of the allele fraction. From the previous results of CNV identification in our simulation, the deletion region ranged from 10% to 15% of the genome size. Thus, a more accurate model for SNV identification is required.

#### Performance evaluation of SNV analysis by simulation studies

**Figure 4:**
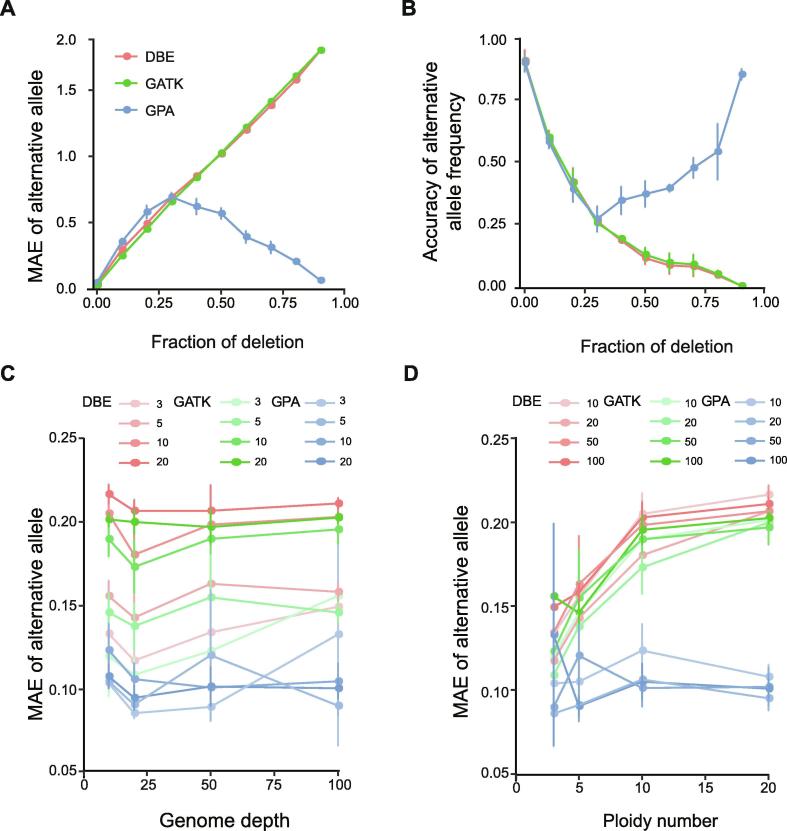
**Evaluation of the GPA package and comparison with other approaches** **A.** The correlation between MAE and deletion fraction in 10 ploidy states with 50× coverage using different methods. MAE rate was calculated under different deletion conditions. Red represents the traditional DBE method, green represents the GATK method, and blue represents the GPA method. **B.** The correlation between accuracy and deletion fraction in 10 ploidy states with 50× coverage using different methods. **C.** The correlation between MAE and sequencing depths. The color gradient of the lines (from light to dark) represents ploidy number of 3, 5, 10, and 20 in the model. **D.** The correlation between MAE and different ploidy numbers. The color gradient of the lines (from light to dark) represents coverage depth in the model spanning 10×, 20×, 50×, and 100×.

To evaluate the performance of SNV analysis of GPA, we use the same simulated data as in the CNV analysis, and firstly assessed the relationship between MAE and deletion fraction in 10 ploidy states with 50× coverage. At the whole genome level, using the GATK and DBE methods, we observed a significant increase in MAE of SNVs when the deletion allele fraction increased. In the same analysis, the GPA method performed better in deletion identification, which corrected most of the CNVs when the deletion fraction was over 0.3, thus resulting in a sharp decrease in MAE (Figure 4A). We also measured the accuracy of the frequencies of alternative alleles and found the accuracy was also significantly increased (Figure 4B). Comparison of results for other states also showed significant improvements in accuracy when CNV was considered in the model (Figures S2 and S3).

We also measured the combined impact of sequencing depth and ploidy number on MAE in SNV analysis. The MAE value showed a steep decrease and kept a constant low level when the depth increased to 20 (Figure 4C). Interestingly, with the increase in ploidy number, the GPA method presented a lower MAE (Figure 4D), which suggested that this method was more suitable for the identification of complex samples than the traditional GATK and DBE method.

### Performance evaluation in complex samples

#### Identification of CNVs and SNVs from Pool-seq data and public metagenomics data

To evaluate the potential of GPA in the discriminant analysis in real datasets, we used two datasets, (1) Pool-seq data from 44 *K. pneumoniae* strains, which could be divided into two groups with the same number (one from 2009, and the other from 2013), and (2) public metagenomics data from three studies on the human gut microbiota [27], [28], [29]. In the GPA package, we integrated genome mapping, CNV and SNV identification, as well as the variant annotation system.

**Figure 5:**
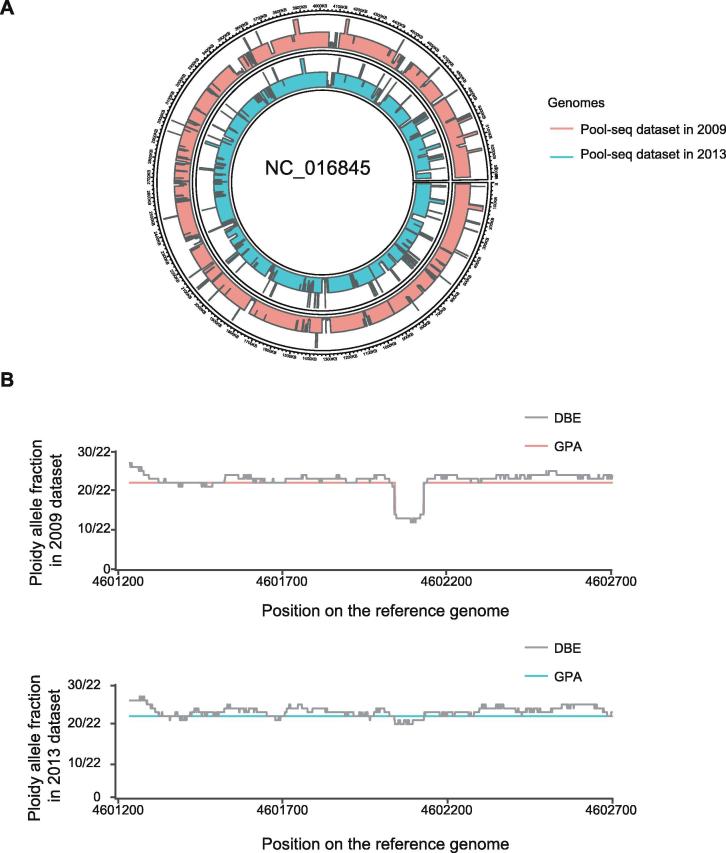
**Comparison of Pool-seq data analyzed by GPA with individually analyzed data from 2009 and 2013 datasets** **A.** Identification of CNVs in Pool-seq data of *K. pneumoniae* in 2009 and 2013 datasets by GPA. The red block represents CNVs found in the 2009 data and the cyan block represents CNVs in the 2013 data. **B.** Detection of deletions in the antimicrobial resistance gene *tolC* in *K. pneumoniae* populations in 2009 and 2013 datasets. The gray line represents the results of DBE analysis; the red and cyan lines represent the results of GPA in 2009 and 2013 populations, respectively.

We evaluated the capability of GPA to discriminate between individual genomes within the Pool-seq data. Using this procedure, we detected 18,131 and 27,819 deletion regions in 2009 and 2013 datasets, respectively, from a total of 9.29 G genome data (∼2000×). Compared to the reference genome, our pipeline observed 10.3% and 10.6% of genomic deletion regions in the data from 2009 and 2013, respectively. Due to the lack of comparable approaches to identify bacterial SNVs and CNVs in complex data, we compared these deletions to the deletion regions which we identified in the alignments of the individual genome to the reference genome data. These deletions covered 23.02% of deletion regions which were observed in the individual genome from 2009 and 60.05% of deletion regions observed in the individual genome from 2013. We also identified 1154 and 216 duplication regions in the dataset from 2009 and 2013, respectively (Figure 5A). Compared to the number of identified deletions in the genome, the number of duplications was limited. SNVs were also identified in these two separate datasets. We observed 692,368 and 272,169 SNV sites in the dataset from 2009 and 2013, respectively, which were similar to the SNV detection by GATK (698,528 and 275,174 sites, respectively). Further analysis of the SNV site coverage to actual genome data presented similar coverage of 79% in the dataset from 2009, but with different MAE rates. In datasets from 2009 and 2013, the MAE rates were 2.00 and 1.78, respectively, when using GATK, which were 1.43 and 1.40, respectively, when using GPA, indicating a lower error rate with the GPA package.

We further evaluated the ability of GPA to discern SNVs within the Pool-seq data by comparing results obtained using GPA and GATK. In the different genetic sites observed by individual genome analysis on the comparison of 2009 with 2013 data, GPA identified 128,803 SNVs. Among them, GATK failed to predict 6675 SNV sites. For example, all six SNV sites in KPHS_45500 were not properly detected by GATK, because there was a 203-bp deletion in 10 of the 22 genome data from 2009 (Table 1). This result suggested that the use of GATK in metagenomics or Pool-seq data from bacteria still has limitations, although it is very effective for data from humans and other mammals.

**Table 1:** **The discrimination of SNVs using GPA and GATK**

**Mutation**	**2009** **Ref allele**	**2009** **Alt allele**	**2009** **Deletion**	**2013** **Ref allele**	**2013** **Alt allele**	**2013** **Deletion**	**Fisher *P* value**	**Method**
Putative glycoside hydrolase	11	2	9	14	6	2	0.034679	True
KPHS_45500:exon1:c.A1518G:p.I506M	10	4	8	16	6	0	0.006104	GPA
	16	6	0	16	6	0	1	GATK

Putative glycoside hydrolase	9	3	10	15	5	2	0.031614	True
KPHS_45500:exon1:c.A1012G:p.K338E	10	4	8	17	5	0	0.005152	GPA
	15	7	0	17	5	0	0.73604	GATK

Putative glycoside hydrolase	11	3	8	10	10	2	0.027615	True
KPHS_45500:exon1:c.T853G:p.C285G	9	4	9	10	12	0	0.00092	GPA
	15	7	0	10	12	0	0.223143	GATK

Putative glycoside hydrolase	10	2	10	19	1	2	0.009187	True
KPHS_45500:exon1:c.A583G:p.I195V	11	2	9	20	2	0	0.001246	GPA
	19	3	0	20	2	0	1	GATK

Putative glycoside hydrolase	4	8	10	13	7	2	0.006573	True
KPHS_45500:exon1:c.G325A:p.E109K	7	6	9	13	9	0	0.003005	GPA
	12	10	0	13	9	0	1	GATK

Putative glycoside hydrolase	10	2	10	19	1	2	0.009187	True
KPHS_45500:exon1:c.A307G:p.T103A	12	1	9	21	1	0	0.001403	GPA
	21	1	0	21	1	0	1	GATK

Last, we expanded the application of the GPA package to metagenomics data in order to identify dynamic changes in gene frequencies within a given genome. The GPA package treated metagenomics data as multi-strain pooled samples. To obtain high enough coverage for the detected species, we evaluated our ability to identify SNVs and CNVs in the dominant species. Using MetaPhlAn2 [30], we found that the dominant species in 15 samples was *Prevotella copri*, with particularly high proportions in the MH0001, MH0005, and MH0018 samples. Using the *P. copri* reference genome (ASM15793v1), we applied the reference-based GPA package to identify CNVs, SNVs, and indel mutations in the three samples. We identified 608, 1245, and 1290 SNV sites in the three samples compared with the *P. copri* reference. Moreover, we identified totally 1,192,437-bp deletion regions and 26,593-bp duplication regions on MH0001, 1,106,144-bp deletion regions and 353,830-bp duplication regions in MH0005, as well as 1,068,700-bp deletion regions and 12,581-bp duplication regions on MH0018. Here, limited by the complexity of the computation, we optimized the number of mixed strains in the same species (N) to 20 in order to balance the precision and efficiency, since the running time will exponentially increase as N increases in GATK. Although we cannot evaluate all the deletions, duplications, and insertions, we compared the number of resistance genes identified by Resistance Gene Identifier [31] to what was identified by BLAST in a previous report. In MH0001, MH0005, and MH0018, respectively, there were 40, 45, and 44 previously identified resistance genes [27]. Among them, 5, 5, and 4 genes were from *P. copri*, which matched to the reference genome ASM15793v1. In our GPA package, we identified 1, 2, and 3 overlapping genes, respectively. We identified 15 AMR genes in reference genome ASM15793v1. In addition, we also identified another 10, 9, and 9 antibiotic resistance genes in these metagenomics data (Table S1). Moreover, we identified the deletion and duplication regions, and calculated the specific fraction of modified alleles in these regions. Among these AMR genes, 4 genes, *EF-Tu*, *rpoB*, *gyrA*, and *ileS*, performed antibiotic resistance through mutations, none of which were identified in previous BLAST pipelines.

#### The application of the GPA package to population comparisons

To better apply the GPA package in the comparison of two groups, we constructed a complete pipeline for genome annotation using reference genome data. In a comparison of two Pool-seq data, *K. pneumoniae* strains from 2009 and 2013, we screened the whole genome and found significantly different genomic regions, including 184,654-bp CNV regions and 128,803 SNVs. Among these CNVs and SNVs, 79.7% and 90.9%, respectively, were from coding regions, which was similar to the percentage of coding regions in *K. pneumoniae*. Then, using an automated model of ANNOVAR [32] with the reference genome, we annotated SNVs with 8919 non-synonymous and 51,053 synonymous sites. Thus, we can quickly and easily learn the biological meaning of these differences since this is an unusual pipeline in its ease of application for the identification of gene function within large, complex datasets.

This package also permitted automated annotation of SNV sites and CNV regions identified by our pipeline in metagenomics data with the *P. copri* reference genome (ASM15793v1). In the MH0001 dataset, 608 SNV sites were identified; 1245 SNV sites were found in MH0005, and 1290 SNV sites were found in MH0018. Of these mutations, 43 sites in MH0001, 78 sites in MH0005, and 64 sites in MH0018 were identified as functional SNV sites, including nonsynonymous mutations and stop-gain mutations. The mutations were located in 5 genes across the three samples. The CNV regions were annotated to 7 genes in MH0001, 10 genes in MH0005, and 7 genes in MH0018.

#### Validating the impact of mutations on virulence and antimicrobial resistance genes

The GPA model for the estimation of gene abundance was applied to Pool-seq datasets from 2009 and 2013 to evaluate the dynamic changes in the frequencies on the functional gene. We compared the frequency of the virulent genes (*rmpA* and *rmpA2*) and antibiotic resistant gene (*tolC*) to two Pool-seq datasets. Among them, *rmpA* and *rmpA2* are genes recently validated in CR-HvKP, whose percentage was recently reported as rapidly elevated in 2013 by our team [33]. Using the GPA package, we estimated that the *rmpA* gene was increased from 7 copies in 2009 to 13 copies in 2013. Also, we estimated that the copy number of *rmpA2* was increased from 10 to 13. This increase was consistent with our observations in individual genomes and phenotypes, although the copy number was not predicted beforehand [33]. *tolC* is a common protein subunit among many multidrug efflux complexes in Gram-negative bacteria. This gene was observed to have a partially deleted region of ∼90 bp in 10 strains in the 2009 dataset, but only 2 strains in the 2013 dataset. Using the GPA package, we detected a deleted region in the gene in the 2009 data but failed to detect it in the 2013 data (Figure 5B). In a comparison of differences in estimated copy number between the two datasets, GPA also identified a significant increase of the strain without deletions in 2013, which suggested that potential antibiotic resistance might be elevated by the active efflux of the drug from the periplasmic entrance using the efflux complex (Table S2). The estimated result by GPA was also more stable than with DBE in our analysis of a single gene (Figure 5B).

We also estimated the antibiotic gene frequency in metagenomics data. The *mexF* gene, a resistance-nodulation-cell division (RND) multidrug efflux transporter, was present in 0.6% deletion regions in MH0001, in 65.7% of the duplication regions in MH0005, and conserved without mutation in MH0018. However, lacking of phenotypic and individual genomic data, we could not well evaluate our accuracy in this dataset.

## Discussion

Metagenomics tools provide powerful insights into the study of populations of clinical or pathogenic microbiota [34]. To fully exploit its potential in the discrimination of genetic variants, including CNVs and SNVs, data analysis tools and algorithms are needed for detecting dynamic changes in complex communities, and evaluating their effects on toxin production, antimicrobial resistance, and other phenotypes [27]. The GPA package is a Bayesian approach for estimation of microbial genetics modal assignments, especially useful in complex clinical samples (Table S3). Using individual genomes and Pool-seq genomic data, we assessed the accuracy, sensitivity, and stability in a uniform model. Compared to the traditional method, this model efficiently decreased the false discovery rate (FDR) of CNV and MAE of SNV in genotype calling. Considering that SNV and CNV profiles were two major factors in the analysis of metagenomic samples, and given the limited general methods that can estimate their distribution and relative abundance, we propose that Bayesian estimation could improve the discrimination of CNVs and SNVs between populations. In addition, we have constructed a GPA package with a complete workflow to analyze Pool-seq data from clinical or environmental samples, which will help researchers to automatically align reads to a selected reference, identify CNV and SNV variants, calculate the accuracy of the allele fraction, and annotate these variants.

In the GPA package, we have improved three aspects of the genotyping of bacterial genomes. First, the GPA package uses the Bayesian model to detect CNV allele fraction, which decreases FDR and MAE significantly compared to traditional DBE. Second, the SNV allele fraction is modified according to the CNV allele fraction, to correct the bias caused by the deletion allele. The accuracy of genotyping provides a foundation for further genotype–phenotype study. Third, this is a complete package containing the major workflow used in microbial assignment in complex samples, and optimized with Pool-seq and whole genome/metagenomics sequence data from clinical settings, which could be used in hospital-based epidemiological molecular surveillance.

A limitation of GPA is the selection of the represented reference sequences. The integrity and complexity of the references may influence the statistical estimation of deletions, insertions, duplications, and translocations, which occur in bacterial populations. A sliding ploidy number used in the model will also extend the area of its application, instead of the assumed ploidy number given in our multi-ploidy model. For its application in metagenomics, we assume the sample is a multi-ploidy model, which may not be true in actual samples. The computing time increases exponentially with the increase in ploidy number, which also limits the application in metagenomics sequence data since we cannot increase the ploidy number too much to obtain an approximation in true metagenomics samples. Metagenomics and Pool-seq are major breakthrough technologies in clinical identification. Bayesian estimation provides an algorithm for accurate and thorough study of the epidemiology, distribution, and pathogenic potential of the reference strain. This approach will also provide considerable insight into other characteristics of individual strains, such as sequence type and gene evolution.

## Materials and methods

### Materials used for GPA evaluation

#### Whole genome sequencing and antibiotic resistance assays for 44 isolates of *K. pneumoniae*

We randomly selected 44 *K. pneumoniae* isolates obtained in 2009 and 2013 (22 in each year) (Table S2). The genomic DNA from these isolates was extracted and purified using a QIAamp DNA Mini Kit (QIAGEN, Hilden, Germany). Then we prepared 500 bp libraries from the genomic DNA of each isolate using NEBNext Ultra DNA Library Prep Kit for Illumina. The libraries were sequenced on the Illumina Hiseq 2500 platform to generate 125 bp paired-end reads (Illumina, San Diego, CA, USA) according to the Illumina manual. The sequence data were deposited in the GenBank database (Accession number: SRP075790). The MICs of these isolates tested with 10 antibiotic agents from eight categories were determined using the broth microdilution method (Table S2).

#### Simulated data of individual genome sequence data and Pool-seq data

We prepared simulated data to evaluate the accuracy of the GPA package for different coverage depths and ploidies by sampling the data from individual ∼150× shotgun reads. NC_016845 was used as reference genome. The BAM files were sampled with the DownsampleSam program in PICARD tools (v1.141). Each raw sample was down-sampled to 3 independent replicates with different coverage depths (5×, 10×, 15×, 20×, 50×, 100×). For the given depth of each sample, we randomly selected the simulated reads from other bacteria to generated the pooled sequence data with 3, 5, 10, 20 mixed sample data.

#### Evaluation of simulated data

We mapped the simulated and original data from FASTQ files to the reference genome using BWA-mem with default parameters, and then removed unmapped reads, low quality reads (Q < 30), and multiple mapping reads to obtained the BAM file. These BAM files were used to calculate the sequencing depth and coverage for each genome. GPA and DBE approaches were evaluated using simulated Pool-seq data and individual genome shotgun sequence data.

#### Pool-seq data and metagenomics data

We mixed DNA from the aforementioned 22 *K. pneumoniae* isolates from both years using average concentrations to make a uniform level of DNA. We then constructed 2 Pool-seq libraries using NEBNext Ultra DNA Library Prep Kits for Illumina and then sequenced on an Illumina Hiseq 2500 platform. Metagenomics data were downloaded from the BGI website (http://gutmeta.genomics.org.cn/), with accession number from MH0001 to MH0020 according to previously described methods [27]. These two datasets were also used to evaluate the discrimination of GPA in simulated data.

### Description of the model for CNV detection

#### Likelihood ploidy state at each position is correct

For the Pool-seq data of n samples, we considered 2∗n+1 ploidy states in this position. In each position i, the genotype $\theta_{i}$ could be expressed as

$$\theta_{i}=\left\{\begin{array}{cc} 0/n, & \text{if this position is deleted in}\;n\;\text{ploidy}\\ 1/n, & \text{if this position is deleted in}\;n-1\;\text{ploidy}\\ \; & \vdots \\ (n-1)/n & \text{if this position is deleted in}\;\;1\;\text{ploidy}\\ 1, & \text{if this position is normal in}\;n\;\text{ploidy}\\ (n+1)/n & \text{if this position is duplicated in}\;\;1\;\text{ploidy}\\ \; & \vdots \\ (2n-1)/n & \text{if this position is duplicated in}\;n-1\;\text{ploidy}\\ 2 & \text{if this position is duplicated in}\;n\;\text{ploidy} \end{array}\right)$$

The Poisson model has been widely employed for analyzing sequence data as well as for differential gene expression [35]. However, the Poisson distribution assumes that the variance of reads is equal to its mean, and with only one parameter. The negative binomial model permitted additional parameters that could be used as an alternative to model the larger variance, for example in CNV detection and Pool-seq data analysis [36]. In the GPA model, we modeled the depth of each position by a negative binomial distribution,

$$P\left(d_{i}|\theta_{i}\right)=nbinom\left(d_{i},\kappa_{\theta_{i}},\mu_{\theta_{i}}\right)=\frac{\Gamma (d_{i}+\kappa_{\theta_{i}})}{\left(d_{i}\right)!\Gamma \left(\kappa_{\theta_{i}}\right)}*{\left(\frac{\kappa_{\theta_{i}}}{\kappa_{\theta_{i}}+\mu_{\theta_{i}}}\right)}^{\kappa_{\theta_{i}}}*{\left(\frac{\mu_{\theta_{i}}}{\kappa_{\theta_{i}}+\mu_{\theta_{i}}}\right)}^{d_{i}}$$

The dispersion parameter $\kappa_{\theta_{i}}$ was set as a global parameter κ for each single experiment. The mean parameter $\mu_{\theta_{i}}$ was the expected depth of each site, which should be proportional to the ploidy$\theta_{i}$, *i.e.*, $\mu_{\theta_{i}}=a*\theta_{i}$.

We assumed that most positions were normal ploidy, which means that the main peak of depth distribution is contributed by $\theta_{i}=1$. To cover more than 95% of the non-zero regions in sequencing depth, we defined the range of 1/2 to 3/2 as the median depth. This peak value works well in single genome (Kpn24 genome as an example), 3 pooled genomes (Kpn12, Kpn14, and Kpn32), and true metagenomics data (MH0005) in our study (Figure S4). We applied the maximum likelihood method to fit the main peak of observed depth distribution, estimated dispersion parameter κ, and the coefficient a. The likelihood was calculated with $\mu_{\theta_{i}}$. For $\theta_{i}=0$, the $\mu_{\theta_{i}}$ was set as 1 since there are a few false positive reads in total deletion regions.

#### Iteratively updating the prior of the ploidy state

Prior probabilities of each ploidy states were estimated with an iterative algorithm. We started from equal probabilities,

$$P\left(\theta_{i}\right)=\frac{1}{\left(2n+1\right)}$$

The ploidy state of each position in the data was determined by the highest posterior probability, which was calculated as

$$P\left(\theta_{i}|d_{i}\right)\propto P\left(d_{i}|\theta_{i}\right)*P\left(\theta_{i}\right)$$

After determining the ploidy state of each observed position, the prior probabilities of ploidy state $P(\theta_{i})$ were updated. The repeated process will continue until $P(\theta_{i})$ convergence to a stable state. The genotyping quality score was calculated as a Phred score of the maximum likelihood to qualify the confidence of the results.

### SNV allele fraction correction of the deletion genotyping data

For the majority of bacterial genomes, deletions are widely distributed, potentially as high as 10% compared to the reference genome. The effect of CNVs cannot be ignored in SNV detection. For a particular position in the genome, ignoring a deletion would lead to an over-estimated allele count of alternative alleles, while ignoring a duplication might lead to a decreased allele fraction.

In our pipeline, we applied the UnifiedGenotyper in GATK software (version 3.4) [17] to detect bi-allelic SNVs. The parameter “-ploidy” for each position was set according to the CNV result.

### Mutation annotation and population analysis

Functional annotation of mutations is important in the research of a bacterial population. However, for the diversity of bacterial genomes, there is not a general method for a mutation annotation pipeline. Breseq is not flexible in the annotation of a custom mutation list and genes. We have developed a pipeline for mutation annotation based on ANNOVAR (version 2017-06-01) [32]. ANNOVAR software is designed for human mutation annotation, and also provides solutions for novel genomes and gene annotations. A genome FASTA file and a gene annotation file in GenePred format were required for file preparation. Additional programs for file format conversion were provided in our software package. The AMR gene was annotated by the Resistance Gene Identifier pipeline (RGI v3.1.1) using the Comprehensive Antibiotic Resistance Database (CARD v1.1.7) [31].

In the comparison mode, the GPA package provided information on the difference in allele frequency between two populations. Mutations with an allele frequency were listed in the results, with the statistical significance calculated by Fisher’s exact test [37].

## Authors’ contributions

CC and HZ conceived the study. CC and JL designed the package. JL built the package and performed the statistical analysis. PD participated in the microbial gene annotation and population analysis. YY participated in the statistical model building. YZ and CS carried out the bacterial isolation, culturing and sequencing. CC and JL drafted the manuscript. All authors discussed the results, commented on the manuscript, and approved the final version for publication.

## Competing interests.

The authors have declared no competing interests.

## Supplementary material

Supplementary Figure S1 **A comparison of the distribution of ploidy allele fractions to coverage depths in original data, GPA and DBE analyses** The color panel from blue to red represents the 7 ploidy allele fractions from deletion to duplication, the same as in Figure 2A.

Supplementary Figure S2 **The MAE calculated for different deletion fractions at simulated coverage depths** Red represents the traditional DBA method, green represents the GATK method, and blue represents the GPA method.

Supplementary Figure S3 **Calculation of accuracy for different deletion fractions and simulated depths** Red represents the traditional DBA method, green represents the GATK method, and blue represents the GPA method.

Supplementary Figure S4 **Measurement of the cut-off for peak values in single genome data (A), pooled dataset of three genomes (B), and metagenomics data (C)** The green line represents the actual depth distribution for the genome sequence, while the cyan line represents the fitted negative binomial distribution. The shadow region represents the fitted curve for a negative binomial distribution.
