# Supplementary figures and images for: GPA: A Microbial Genetic Polymorphisms Assignments Tool in Metagenomic Analysis by Bayesian Estimation
Source: Genomics Proteomics Bioinformatics. 2019 Apr 23;17(1):106–17. doi: 10.1016/j.gpb.2018.12.005 (PMC6520909; doi:10.1016/j.gpb.2018.12.005)

### A Original

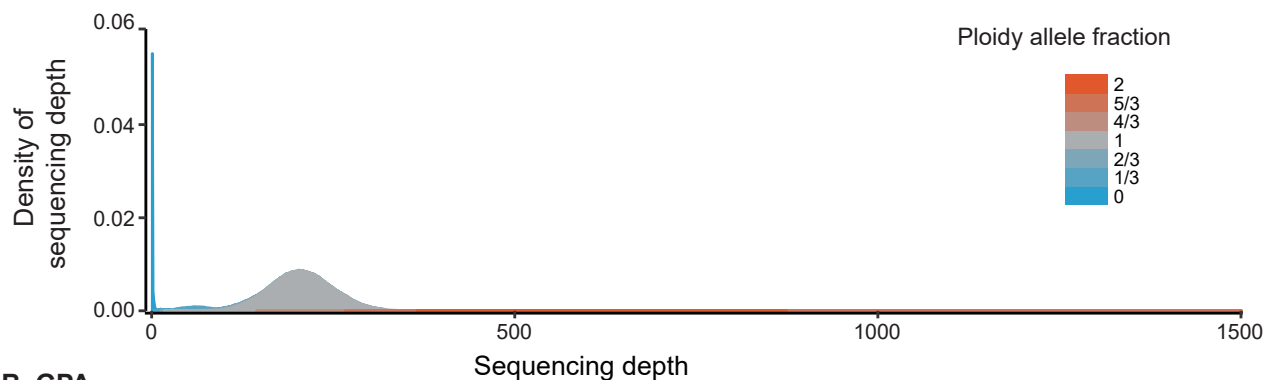

### B GPA

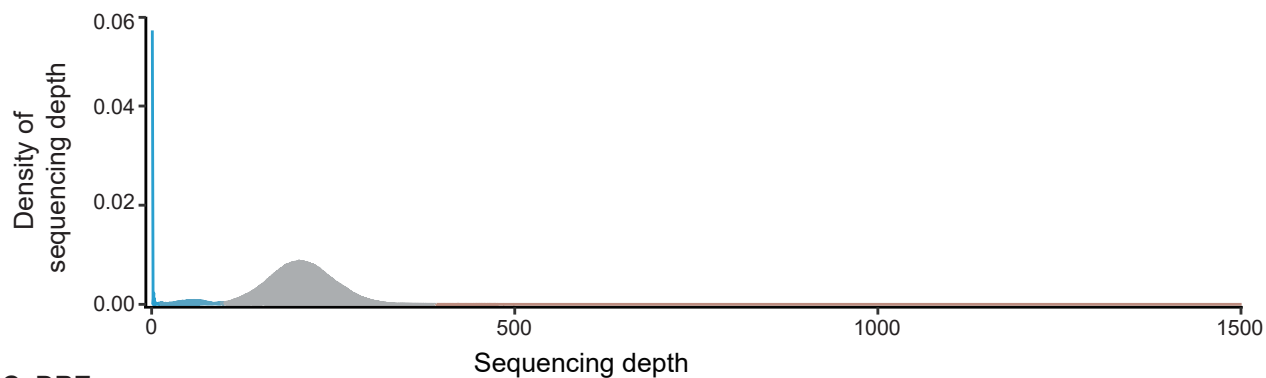

### C DBE

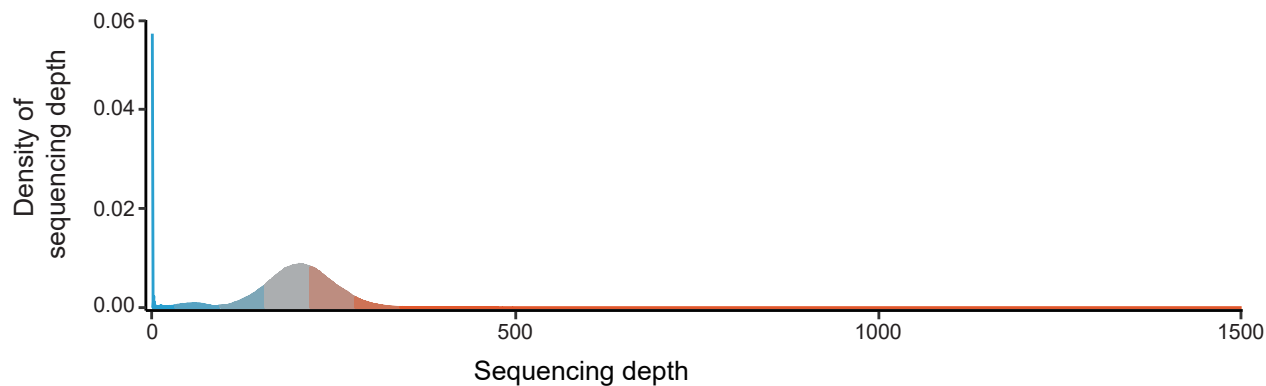

Supplement: Supplementary Figure S1 — A comparison of the distribution of ploidy allele fractions to coverage depths in original data, GPA and DBE analyses The color panel from blue to red represents the 7 ploidy allele fractions from deletion to duplication, the same as in Figure 2A. [file mmc1.pdf]

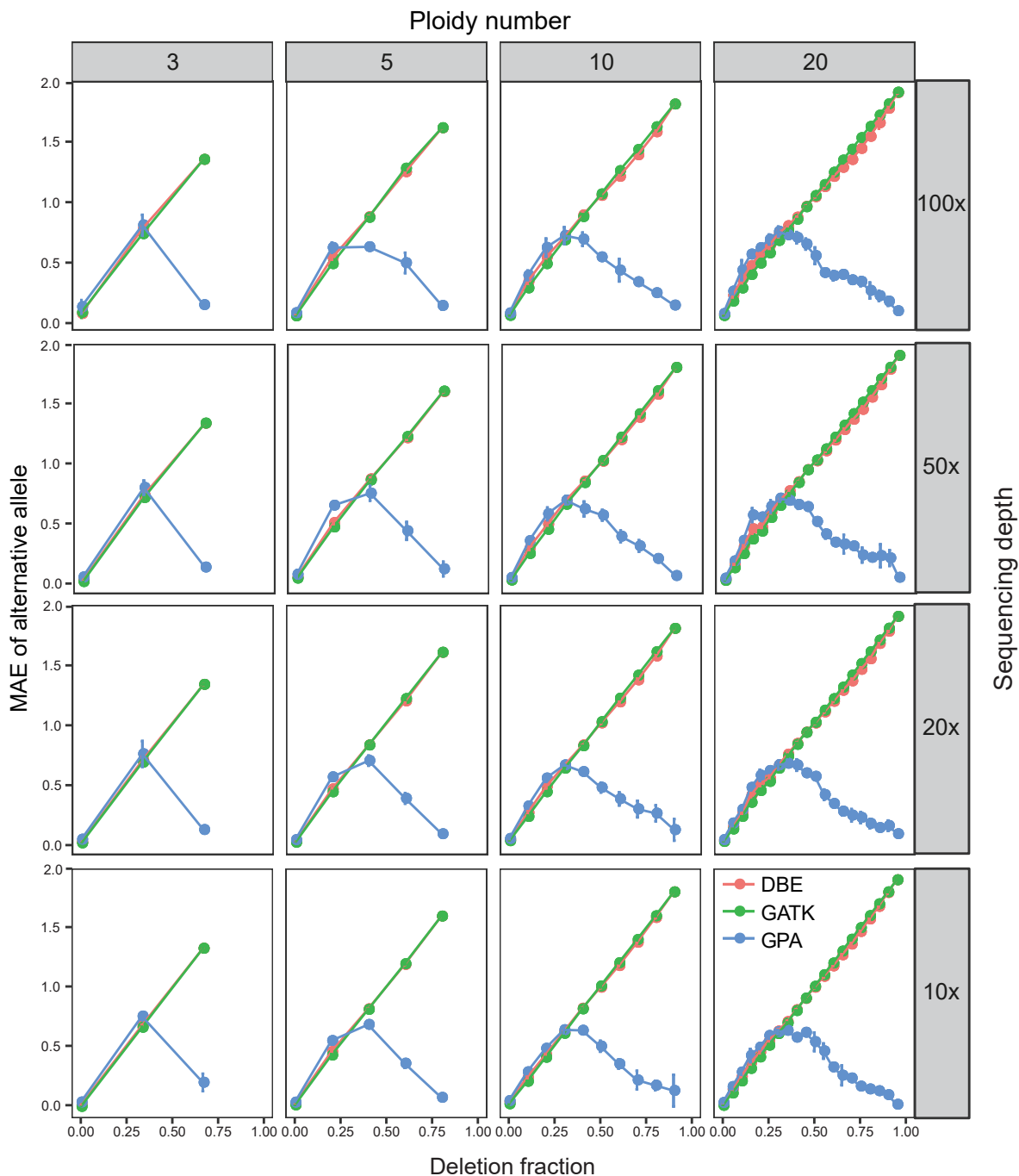

Supplement: Supplementary Figure S2 — The MAE calculated for different deletion fractions at simulated coverage depths Red represents the traditional DBA method, green represents the GATK method, and blue represents the GPA method. [file mmc2.pdf]

# Ploidy number

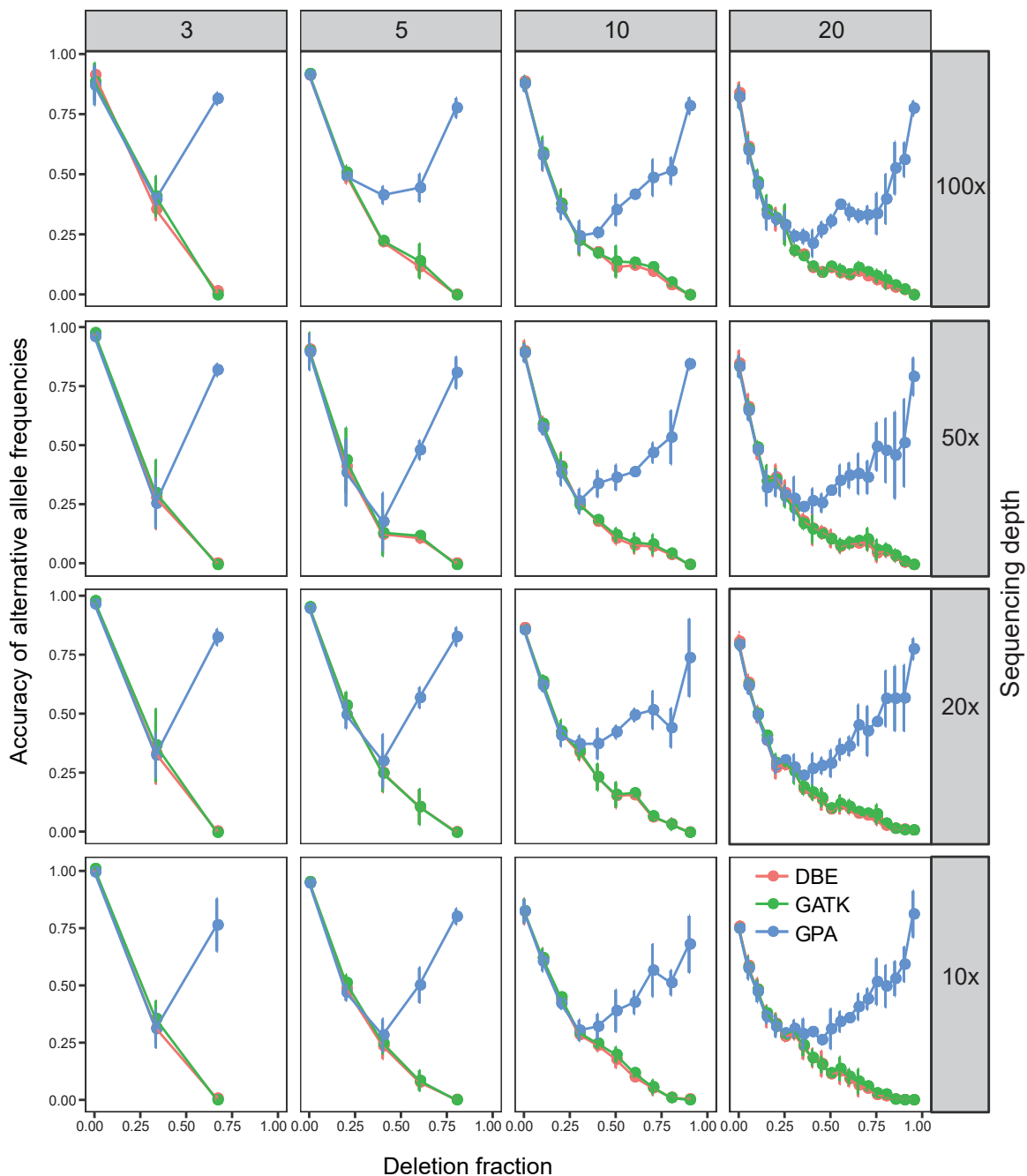

Supplement: Supplementary Figure S3 — Calculation of accuracy for different deletion fractions and simulated depths Red represents the traditional DBA method, green represents the GATK method, and blue represents the GPA method. [file mmc3.pdf]

**A Single genome**

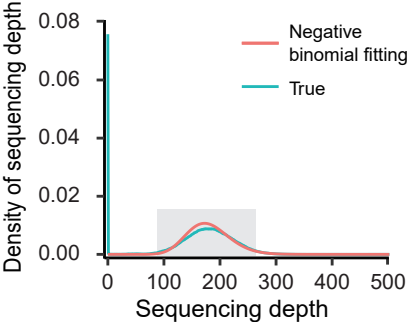

**B Three pooled genomes**

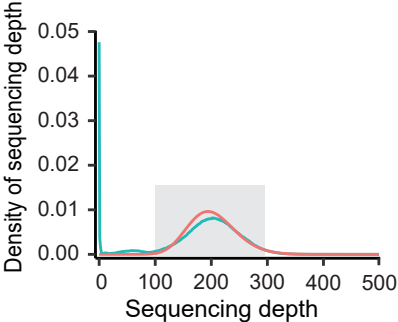

**C Metagenomic sequencing**

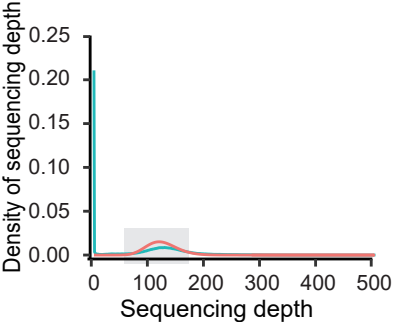

Supplement: Supplementary Figure S4 — Measurement of the cut-off for peak values in single genome data (A), pooled dataset of three genomes (B), and metagenomics data (C) The green line represents the actual depth distribution for the genome sequence, while the cyan line represents the fitted negative binomial distribution. The shadow region represents the fitted curve for a negative binomial distribution. [file mmc4.pdf]
