# Supplementary material for: GPA: A Microbial Genetic Polymorphisms Assignments Tool in Metagenomic Analysis by Bayesian Estimation
Source: Genomics Proteomics Bioinformatics. 2019 Apr 23;17(1):106–17. doi: 10.1016/j.gpb.2018.12.005 (PMC6520909; doi:10.1016/j.gpb.2018.12.005)
Supplement: Supplementary Table S1 [file mmc5.docx]

**Table S1 The antimicrobial resistance genes identified from the metagenomics data**

| **Gene symbol^*^** | **BLAST** | | |  | **GPA package** | | |
| --- | --- | --- | --- | --- | --- | --- | --- |
|  | **MH0001** | **MH0005** | **MH0018** |  | **MH0001** | **MH0005** | **MH0018** |
| bl2e_cepa | 9.28E−06 | 2E−06 | 4.63E−05 |  | 0 | 0 | 0 |
| ceoB | 2.1E−05 | 9.66E−06 | 0.000104 |  | 1 | 68.1% dup | 1 |
| mdtK | 0 | 4.27E−07 | 0 |  | 1 | 1 | 1 |
| cata11 | 9.93E−06 | 3.34E−06 | 1.01E−06 |  | 0 | 0 | 0 |
| cata13 | 1.65E−06 | 0 | 0 |  | 0 | 0 | 0 |
| tetQ | 7.91E−06 | 2.08E−06 | 0.001386 |  | 0 | 0 | 29.4% dup |
| pbp2 | 0 | 0 | 0 |  | 1 | 1 | 1 |
| mexF | - | - | - |  | 0.6% del | 65.7% dup | 1 |
| pmrE | - | - | - |  | 83.7% del | 45.6% del | 67.8% del |
| mfd | - | - | - |  | 1 | 1 | 1 |
| desR | - | - | - |  | 1 | 36.5% dup | 0.1% del |
| EF-Tu | - | - | - |  | 1 | 95.4% dup | 2.6% del |
| rpoB | - | - | - |  | 0.2% del | 50.7% dup | 0.2% del |
| gyrA | - | - | - |  | 0.8% del | 27.9% dup | 1 |
| ileS | - | - | - |  | 1.3% del | 81.9% dup | 1 |

*Note*: *, Gene symbol from the CARD database v1.1.7. Del, Deletion; Dup, Duplication.
