# Supplementary material for: GPA: A Microbial Genetic Polymorphisms Assignments Tool in Metagenomic Analysis by Bayesian Estimation
Source: Genomics Proteomics Bioinformatics. 2019 Apr 23;17(1):106–17. doi: 10.1016/j.gpb.2018.12.005 (PMC6520909; doi:10.1016/j.gpb.2018.12.005)
Supplement: Supplementary Table S2 [file mmc6.docx]

**Table S2 The character of antimicrobial resistance of the 44 isolated *K. pneumonia***

| **Isolate** | **Year** | **Cephems** | | | |  |
| --- | --- | --- | --- | --- | --- | --- |
|  |  | **Cefepime** | **Ceftazidime** | **Ceftriaxone** | **Cefuroxime** |  |
| Kpn1 | 2009 | S | S | R | R |  |
| Kpn2 | 2009 | S | S | R | R |  |
| Kpn3 | 2009 | S | R | R | R |  |
| Kpn4 | 2009 | S | S | I | R |  |
| Kpn5 | 2009 | S | S | S | R |  |
| Kpn6 | 2009 | S | S | S | S |  |
| Kpn7 | 2009 | S | S | S | R |  |
| Kpn8 | 2009 | S | S | S | S |  |
| Kpn9 | 2009 | S | S | R | R |  |
| Kpn10 | 2009 | S | S | R | R |  |
| Kpn11 | 2009 | S | S | R | R |  |
| Kpn12 | 2009 | S | S | R | S |  |
| Kpn13 | 2009 | S | S | R | R |  |
| Kpn14 | 2009 | S | S | S | S |  |
| Kpn15 | 2009 | S | S | S | S |  |
| Kpn16 | 2009 | S | S | S | S |  |
| Kpn17 | 2009 | S | S | S | S |  |
| Kpn18 | 2009 | S | S | R | R |  |
| Kpn19 | 2009 | S | I | R | R |  |
| Kpn20 | 2009 | S | S | R | R |  |
| Kpn21 | 2009 | S | S | R | R |  |
| Kpn22 | 2009 | S | R | R | R |  |
| Kpn23 | 2013 | S | S | R | R |  |
| Kpn24 | 2013 | S | S | R | R |  |
| Kpn25 | 2013 | S | R | R | R |  |
| Kpn26 | 2013 | S | S | R | R |  |
| Kpn27 | 2013 | S | S | S | S |  |
| Kpn28 | 2013 | S | R | R | R |  |
| Kpn29 | 2013 | S | R | R | R |  |
| Kpn30 | 2013 | S | R | R | R |  |
| Kpn31 | 2013 | S | S | R | R |  |
| Kpn32 | 2013 | S | S | R | R |  |
| Kpn33 | 2013 | S | S | S | S |  |
| Kpn34 | 2013 | S | R | S | I |  |
| Kpn35 | 2013 | S | S | R | R |  |
| Kpn36 | 2013 | S | S | R | R |  |
| Kpn37 | 2013 | S | R | R | R |  |
| Kpn38 | 2013 | S | R | R | R |  |
| Kpn39 | 2013 | S | R | R | R |  |
| Kpn40 | 2013 | S | I | R | R |  |
| Kpn41 | 2013 | S | S | R | R |  |
| Kpn42 | 2013 | S | S | R | R |  |
| Kpn43 | 2013 | S | I | R | R |  |
| Kpn44 | 2013 | S | I | R | R |  |
| **Isolate** | **Year** | **Penicillins** | **Penems** | | **Aminoglycosides** |  |
|  |  | **Piperacillin** | **Imipenem** | **Amikacin** | **Meropenem** |  |
| Kpn1 | 2009 | R | S | R | S |  |
| Kpn2 | 2009 | R | S | R | S |  |
| Kpn3 | 2009 | R | R | R | R |  |
| Kpn4 | 2009 | R | S | R | S |  |
| Kpn5 | 2009 | R | S | S | S |  |
| Kpn6 | 2009 | R | S | S | S |  |
| Kpn7 | 2009 | R | S | S | S |  |
| Kpn8 | 2009 | R | S | S | S |  |
| Kpn9 | 2009 | R | S | S | S |  |
| Kpn10 | 2009 | R | S | S | R |  |
| Kpn11 | 2009 | R | R | S | R |  |
| Kpn12 | 2009 | R | R | S | R |  |
| Kpn13 | 2009 | R | R | S | R |  |
| Kpn14 | 2009 | R | S | S | S |  |
| Kpn15 | 2009 | R | S | S | S |  |
| Kpn16 | 2009 | R | S | S | S |  |
| Kpn17 | 2009 | R | S | S | S |  |
| Kpn18 | 2009 | R | S | S | S |  |
| Kpn19 | 2009 | R | S | S | S |  |
| Kpn20 | 2009 | R | S | S | S |  |
| Kpn21 | 2009 | R | S | S | S |  |
| Kpn22 | 2009 | R | R | R | R |  |
| Kpn23 | 2013 | R | S | S | R |  |
| Kpn24 | 2013 | R | S | S | S |  |
| Kpn25 | 2013 | R | S | S | S |  |
| Kpn26 | 2013 | R | S | S | S |  |
| Kpn27 | 2013 | R | S | S | S |  |
| Kpn28 | 2013 | R | S | R | S |  |
| Kpn29 | 2013 | R | S | R | S |  |
| Kpn30 | 2013 | R | S | R | S |  |
| Kpn31 | 2013 | R | S | S | S |  |
| Kpn32 | 2013 | R | S | S | S |  |
| Kpn33 | 2013 | R | S | S | S |  |
| Kpn34 | 2013 | R | S | S | S |  |
| Kpn35 | 2013 | R | S | S | S |  |
| Kpn36 | 2013 | R | S | S | S |  |
| Kpn37 | 2013 | R | S | S | S |  |
| Kpn38 | 2013 | R | S | R | S |  |
| Kpn39 | 2013 | R | R | S | I |  |
| Kpn40 | 2013 | R | S | S | S |  |
| Kpn41 | 2013 | R | S | S | S |  |
| Kpn42 | 2013 | R | S | S | S |  |
| Kpn43 | 2013 | R | S | S | S |  |
| Kpn44 | 2013 | R | S | S | S |  |
| **Isolate** | **Year** | **Quinolones** | **Tetracyclines** | **Fosfomycins** | **Lipopeptides** | |
|  |  | **Levofloxacin** | **Tigecycline** | **Fosfomycin** | **PolymyxinB** | **Colistin** |
| Kpn1 | 2009 | S | R | R | S | R |
| Kpn2 | 2009 | S | R | R | R | R |
| Kpn3 | 2009 | R | R | R | R | R |
| Kpn4 | 2009 | R | R | R | S | R |
| Kpn5 | 2009 | S | R | R | R | R |
| Kpn6 | 2009 | S | I | R | S | R |
| Kpn7 | 2009 | S | I | I | S | R |
| Kpn8 | 2009 | S | I | R | S | R |
| Kpn9 | 2009 | R | I | R | R | R |
| Kpn10 | 2009 | S | I | R | S | R |
| Kpn11 | 2009 | S | R | I | S | R |
| Kpn12 | 2009 | S | S | I | S | R |
| Kpn13 | 2009 | S | I | R | R | R |
| Kpn14 | 2009 | S | R | R | S | R |
| Kpn15 | 2009 | S | I | I | S | R |
| Kpn16 | 2009 | S | S | I | S | R |
| Kpn17 | 2009 | S | I | I | S | R |
| Kpn18 | 2009 | S | R | I | R | R |
| Kpn19 | 2009 | S | R | I | S | R |
| Kpn20 | 2009 | S | R | I | R | R |
| Kpn21 | 2009 | S | R | R | S | R |
| Kpn22 | 2009 | R | I | R | R | R |
| Kpn23 | 2013 | S | I | R | R | R |
| Kpn24 | 2013 | R | I | R | S | R |
| Kpn25 | 2013 | R | I | R | R | R |
| Kpn26 | 2013 | R | R | R | S | R |
| Kpn27 | 2013 | R | R | R | R | R |
| Kpn28 | 2013 | R | R | R | R | R |
| Kpn29 | 2013 | R | I | R | R | R |
| Kpn30 | 2013 | R | R | I | R | R |
| Kpn31 | 2013 | S | R | I | S | R |
| Kpn32 | 2013 | S | R | I | S | R |
| Kpn33 | 2013 | S | I | R | S | R |
| Kpn34 | 2013 | S | R | I | S | R |
| Kpn35 | 2013 | S | R | I | R | R |
| Kpn36 | 2013 | S | R | R | R | R |
| Kpn37 | 2013 | R | R | R | R | R |
| Kpn38 | 2013 | R | R | R | R | R |
| Kpn39 | 2013 | R | R | R | R | R |
| Kpn40 | 2013 | S | R | R | R | R |
| Kpn41 | 2013 | S | I | I | S | R |
| Kpn42 | 2013 | S | R | I | R | R |
| Kpn43 | 2013 | S | I | I | R | R |
| Kpn44 | 2013 | S | I | I | R | R |

*Note*: S, susceptible; R, Resistant; I, Intermediate.
