# Supplementary material for: GPA: A Microbial Genetic Polymorphisms Assignments Tool in Metagenomic Analysis by Bayesian Estimation
Source: Genomics Proteomics Bioinformatics. 2019 Apr 23;17(1):106–17. doi: 10.1016/j.gpb.2018.12.005 (PMC6520909; doi:10.1016/j.gpb.2018.12.005)
Supplement: Supplementary Table S3 [file mmc7.docx]

**Table S3 The comparison of software tools for CNV and SNV detection, quantification, and annotation**

|  | **GPA** | **GATK** | **ANNOVAR** | **Breseq** | **BLAST** |
| --- | --- | --- | --- | --- | --- |
| CNV detection | √ |  |  | √ |  |
| SNV detection | √ | √ |  | √ |  |
| SNV quantification | √ | √ |  |  |  |
| CNV quantification | √ |  |  |  |  |
| CNV annotation | √ |  |  | √ | √ |
| SNV annotation | √ |  | √ | √ |  |
| For complex sample | √ | √ |  |  | √ |
